# Angiome rétinien de la maladie de Von Hippel-Lindau

**DOI:** 10.11604/pamj.2014.18.31.4016

**Published:** 2014-05-08

**Authors:** Ryme Abdelkhalek, Nestor Aigbé

**Affiliations:** 1 Service d'Ophtalmologie, Hôpital Militaire d'Instruction Mohamed V, Rabat, Maroc

**Keywords:** Angiome, retina, Von Hippel-Lindau disease, Angioma, rétine, maladie de Von Hippel-Lindau

## Image en medicine

Patient âgé de 28 ans, non consanguin, 2^ème^ d'une fratrie de 4, qui consulte pour une baisse d'acuité visuelle bilatérale. L'examen de l'oeil droit trouve une acuité visuelle limité au compté des doigts. Le fond d'oeil met en évidence 2 hémangiomes capillaires rétiniens (RCH): (A, B). L'examen de l'oeil gauche trouve une acuité visuelle de 4/10 sans correction. Le fond d'oeil révèle de multiples hémangioblastomes non compliqués (C). Le bilan systémique montre la présence d'un Kyste rénal ayant bénéficié d'un traitement conservateur, et une tumeur du pancréas. L’étude moléculaire du gène VHL a montré qu'il est porteur de la mutation c.499 C>T; p.Arg 167 Trp à l’état hétérozygote. Le traitement a consisté en une cryothérapie au niveau de l'oeil droit. L’évolution a été marquée par une récupération fonctionnelle partielle avec la formation d'une membrane fibrovasculaire du pole postérieur (B).

**Figure 1:**
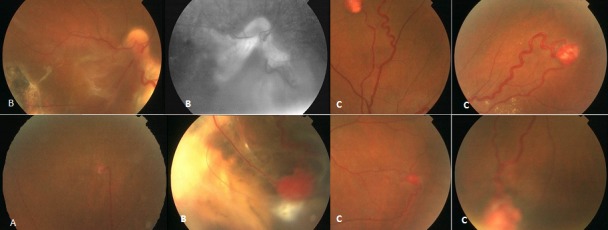
A) Petit hémangiome capillaire rétinien supérieur de l'oeil droit; B) Hémangiome capillaire rétinien de grande taille de l'oeil droit compliqué de décollement séreux rétinien avec cicatrice de cryothérapie; C) 4 Hémangiomes capillaires rétiniens de l'oeil gauche

